# Enhancing Driving Ability in Older Adults through Health Exercises and Physical Activity: A Randomized Controlled Trial

**DOI:** 10.3390/ijerph20196802

**Published:** 2023-09-22

**Authors:** Akihiko Katayama, Ayako Hase, Nobuyuki Miyatake

**Affiliations:** 1 Faculty of Sociology, Shikoku Gakuin University, Zentsuji-shi 765-8505, Kagawa, Japan; 2 Department of Clinical Psychology, Faculty of Medicine, Kagawa University, Miki-cho 761-0701, Kagawa, Japan; hase.ayako@kagawa-u.ac.jp; 3 Department of Hygiene, Faculty of Medicine, Kagawa University, Miki-cho 761-0701, Kagawa, Japan; miyatake.nobuyuki@kagawa-u.ac.jp

**Keywords:** driving ability, healthy exercise, physical activity, randomized controlled trial

## Abstract

The global rise in the aging driving population has heightened concerns about traffic incidents involving this demographic. Beyond transportation, automobiles represent a vital lifeline for older adults, fostering social activities and influencing their health-related quality of life. This study explores improving and sustaining driving ability among older adults with anticipated declines through health-conscious exercises. Sixty-eight participants were randomly allocated into two groups. The exercise-oriented group (E-group) engaged in twelve 90 min health and exercise sessions over twelve weeks, while the control group (C-group) maintained their regular daily routines and did not receive any specific interventions during this period. The focal point of assessment was driving ability, as evaluated by a person using a real car on public roads without using a simulator. Driving ability and physical fitness were assessed before the intervention in both groups. Post-intervention measurements occurred twelve weeks after the initial gauging, encompassing both cohorts. Comparative analysis of pre- and post-intervention changes was executed between the two groups. The E-group demonstrated improved overall driving ability compared to the C-group. The results suggest that healthy exercise and physical activity may maintain and enhance driving ability for older adults.

## 1. Introduction

The global population is aging rapidly [1]. The percentage of the world population aged over 65 years is projected to increase from 10% in 2022 to 16% in 2050 [2]. Countries with aging populations are experiencing a need to take steps to adapt their public programs to accommodate an increasing proportion of older adults [2]. Coping with an aging population has become essential in every country and region [3]. Along with the aging population, the number of older drivers is also increasing [4,5]. Road accidents involving older drivers have become a global concern [6]. It has been reported that older adults have difficulty driving cars because of changes in physical function caused by aging [7,8,9].

In recent years, Japan has grappled with an escalating social concern: the rise of traffic accidents involving older drivers. In 2020, the “Emergency Traffic Safety Measures for Older Drivers” article was published as part of the white paper on traffic safety [10]. In response to the challenge of mitigating accidents attributed to age-related physical decline, Japan’s National Police Agency implemented a voluntary driver’s license return system (license revocation upon application) for older individuals experiencing driving uncertainty due to waning physical capabilities [11]. Additionally, Japan introduced an actual driving test (driving skill test) on 13 May 2022, aimed at drivers aged 75 or above during license renewal, particularly those with recent traffic violations [12].

Although traffic accidents involving older drivers have been discussed in areas where public transportation is scarce, using automobiles is essential for the independent lifestyle of older adults [13,14]. With the current increase in older adults, the increase in traffic accidents caused by elderly drivers has become a social problem. However, driving is not just a means of transportation; older adults must maintain social activities. Efforts are needed to enable older adults to continue driving safely and securely. Ongoing efforts encompass the research and development of secure vehicles tailored to the needs of older drivers [15,16,17], reflecting a “hardware (automobile) approach” to car safety. However, the driver remains pivotal. The “human (driver) approach” is equally crucial in upholding driving ability. Richard et al. emphasized that providing a physical conditioning program to older drivers may help them maintain and improve their driving abilities [18]. This suggests that enhancing physical function can maintain and improve driving ability. Therefore, it is possible to choose healthy exercise and physical activity practices to boost the maintenance of driving ability.

Automobiles are a means of transportation and feature of life that generate social activities for older adults in the community. Safe automobile driving in the community is associated with maintaining a health-related quality of life [19,20]. Regular exercise and physical activity have also been reported to directly improve health-related quality of life [21,22]. Suppose that healthy exercises and physical activity methods influence motor vehicle driving ability maintenance. In this case, more active health exercises and physical activity will lead to an improved health-related quality of life, both indirectly and directly. Driving ability can be considered a mere means of transportation, a means of social activity and participation, and a methodology for maintaining and improving health-related quality of life.

This study aimed to examine the possibility of preserving and improving driving ability by practicing healthy exercise and physical activity among older community members, whose driving abilities are expected to decline.

## 2. Materials and Methods

### 2.1. Participants

We recruited research collaborators by distributing materials to administrative agencies, community centers, etc., with local city and town governments’ cooperation. A recruitment briefing session was held at the local Federation of Senior Citizen Clubs. Advertisements for participants were inserted in local newspapers. We accepted participation applications by mail and telephone for seven days. Participants were required to: (1) hold a regular driver’s license, (2) drive a car daily, and (3) have recent driving concerns. The age criterion was 65 years or older due to the study’s focus on older adults. Health-wise, participants needed no doctor-diagnosed exercise contraindications and independent access to the health class venue. Since we require that participants hold a driver’s license, we determined that they retain vision, hearing, and other physical functions that do not impair their driving ability. However, these criteria are essential to this study. Therefore, individual interviews were conducted by public health nurses to confirm general health status, including the above.

### 2.2. Study Design

**Figure 1:**
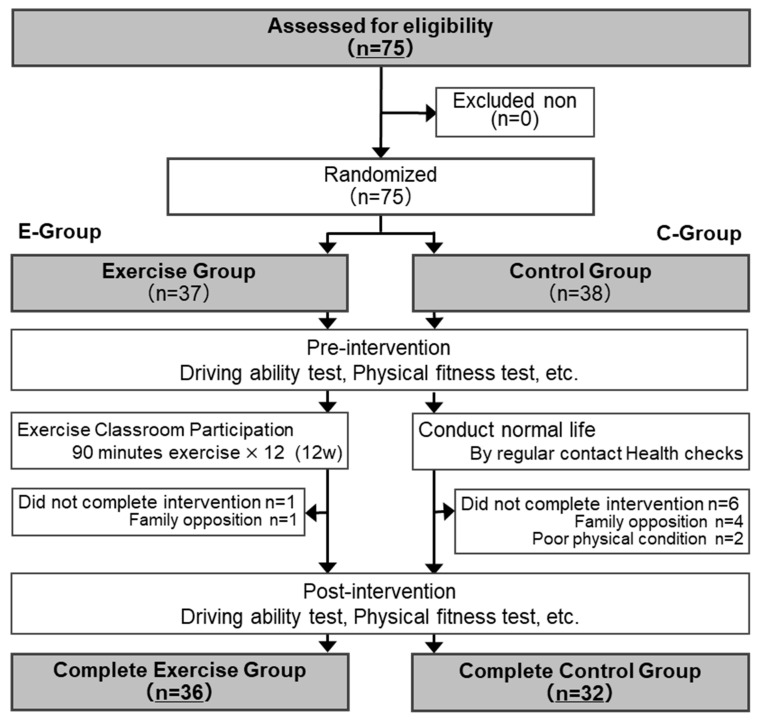
Flow chart of study design.

This study used a randomized controlled trial (RCT) design (Figure 1). Seventy-five participants were assigned to two groups using a stratified block random assignment method based on sex. Randomization involved two categories: a group that participated in the healthy exercise class and practiced healthy exercises (E-group), and a group that did not receive any particular intervention and continued with their daily life routine (C-group). Following random assignment, both groups underwent demographic surveys, driving ability assessments, physical fitness measurements, and other evaluations.

In the E-group, health and exercise classes led by health and exercise instructors were conducted at a public sports facility. Health and exercise classes were held for 90 min weekly for 12 weeks, for 12 sessions. No intervention was offered to the C-group for 12 weeks. Periodic phone calls were made approximately every four weeks to check the health status of the participants. Study participants whose health statuses were continuously confirmed through regular contact were included in the analysis. Twelve weeks after the initial measurement, post-intervention measurements were performed in both groups.

### 2.3. Sample Size

Data on the driving ability measurements of local older adults conducted in the past at driving schools and automobile driving tests were used as reference materials. The sample size was calculated considering a 5-point change in driving skill assessment scores post-intervention, an 8-point standard deviation of the difference, 80% power, and a 5% significance level. A 10% dropout rate was adopted based on previous intervention studies. Finally, 74 participants were included in this study.

### 2.4. Clinical Parameters and Measurements

The sex, age, height (cm), and body weight (kg) of each participant were recorded. Each participant’s body mass index (BMI) was calculated as follows: body weight (kg)/(height (m))^2^.

### 2.5. Driving Ability Measurement

Several studies on driving ability have used driving simulators. However, certified driving test engineers commonly perform driving ability tests legally conducted in Japan. An accredited driving test operator is designated by the Japan Public Safety Commission and issued a driving test operator qualification certificate under Japan’s Road Traffic Law. Furthermore, the tests were conducted at designated driving schools and on “public roads”. In this study, field tests were conducted to measure driving ability.

A challenge in skill tester evaluation is standardizing the calibration or assessment level. To mitigate this, measurement quality was upheld through adherence to Japanese regulations. Technicians responsible for measurement and evaluation were qualified, consistently trained in testing techniques, and reaffirmed scoring criteria before study measurements.

A driving ability test was conducted for each participant at a driving school. After confirming the participant’s identity, the following steps were taken to confirm their health condition: explanation of the measurement schedule; proof of the research measurement course; confirmation of vehicle used. Subsequently, the examiner measured the participants on public roads. The measurement was completed with a recheck of the health condition after completing the measurement. Fifty minutes per participant were allotted for measurement. The measurement was conducted with the participant driving the designated test course for approximately twenty minutes, with the skilled test engineer riding in the passenger seat. All the participants were assessed using the same test course. The same test course was used for the pre-and post-intervention measurements.

The measurements were conducted on public roads, with safety as the foremost consideration across all measurement aspects. The testers agreed that if driving was considered extremely dangerous from a safety management standpoint, the vehicle would stop and the measurements would be terminated at that point. In these instances, retests were not conducted and data from research collaborators who stopped or suspended measurements were excluded from the analysis.

Driving behavior consists of three elements: “cognition”, “judgment”, and “operation [23]”. “Cognition” refers to the perception of the external environment through visual, auditory, and other means. In essence, it recognizes abnormality and danger by checking traffic signs, signals, crosswalks, etc. “Judgment” entails making driving decisions based on the analysis of the external environment. This phase involves the driver selecting actions, such as stopping, moving straight, turning right, etc. “Operation” encompasses executing actions according to the driver’s “decision”. The driver’s actions included steering wheel operations and braking.

The evaluation method was classified into the “recognition”, “judgment”, and “operation” phases, and one point was added when a point reduction item was identified for each phase. During the measurement period, no approaches, such as reminders from the examiner, were implemented even when a point reduction item occurred.

During real-time driving, driving behaviors unfold seamlessly, with the potential for concurrent impact on “cognition”, “judgment”, and “operation”. Alongside individual score evaluations, three categories of composite items were included in the analysis: “cognition” + “judgment” score evaluation, “judgment” + “operation” score evaluation, and “cognition” + “judgment” + “operation” score evaluation.

In this study, the driving ability evaluation was based on a point reduction system; therefore, the higher the number of points for each phase, the lower the driving ability level.

### 2.6. Physical Fitness

Physical fitness was assessed using grip strength (kg), flexibility (cm), one-leg open-eye balance (s), and obstacle walking 10 m (seconds), which were standardized in the New Physical Fitness Test by the Ministry of Education, Culture, Sports, Science, and Technology [24,25]. Additionally, a 30 s chair standing-up test (times) [26,27], five chair standing-up tests (seconds) [28,29], and a timed up-and-go test (seconds) were performed [30,31].

### 2.7. Measures of Executive Functioning and Attentional Functioning

The Trail Making Test (TMT) (TMT-J: Trail Making Test, Japanese version) was used to measure executive and attention functions.

The TMT is an internationally used test method for measuring executive functions [32,33,34]. The reliability and validity of the TMT as a test for attentional function have already been reported. In addition to its ability to measure attentional function, working memory, spatial cognition, and processing speed in a short time and comprehensively, the TMT is also a psychological evaluation method for older drivers’ aptitude, which is the purpose of this study.

The TMT is available in Part A (TMT-A) and Part B (TMT-B). TMT-A comprises numbers, and participants connect them sequentially from “1” to “25” using a writing tool (pencil), with completion time (in seconds) recorded. TMT-B involves numbers “1” to “13” and “A” to “L” in Hiragana (similar to the Japanese alphabet, with letters A, B, C…). The numbers and Hiragana are connected in the same way alternately, with completion time (in seconds) measured.

Participants underwent a practice test before the product test. A shorter completion time indicated superior performance for the TMT.

### 2.8. Statistical Analysis

Continuous variables are presented as mean (standard deviation), and categorical variables are presented as number of persons (percentage, %). The means of the changes before and after the intervention were compared between groups for continuous variables. Unpaired *t*-tests were used for statistical analysis. The significance level was set at *p* < 5% for one-tailed tests. Statistical analyses were performed using “Bell Curve for Excel” (Social Survey Research Information Co., Ltd., Tokyo, Japan).

### 2.9. Response to Blinding

In the driving ability measurements, physical fitness measures, and TMT, the testers performed all tests without knowing the participants’ group. After data collection, the data sheets provided to the data analysts were entirely blinded, and the groups’ names and other information were changed.

### 2.10. Ethics

The study was conducted according to the guidelines of the Declaration of Helsinki. This protocol was registered with the University Hospital Medical Information Network Clinical Trials Registry (registration number: UMIN000044706). This study was approved by the ethical committee of Shikoku Gakuin University, Zentsuji city, Kagawa Prefecture, Japan (approval number: 2021001).

## 3. Results

### 3.1. Clinical Characteristics of Enrolled Participants at Baseline

After a briefing session, consent to participate in the study was obtained from all 75 participants. Stratified random assignments according to sex were conducted. In the E-group, one participant withdrew from the study (due to family opposition to participation in the survey). In the C-group, six participants withdrew from the study (four with family objections to participation and two with physical illness). Following pre-intervention measurements, a 12-week healthy exercise intervention and routine daily life, as well as post-intervention assessments, 36 participants in the E-group and 32 in the C-group completed all measurements and were included in the analysis. The characteristics of the 68 participants are presented in Table 1. The participants’ mean age was 74.9 (SD = 3.8), with 52 patients (76.5%) being female.

Table 2 shows the characteristics of each group after stratifying random block assignments by sex. The E-group had one dropout (one male), resulting in a female ratio of 80.1%. The C-group had six dropouts (six females), with a female ratio of 71.9% (chi-square test: chi-square value = 0.71, *p* = 0.39).

### 3.2. Comparison of the Changes in Parameters between the Two Groups

**Figure 2:**
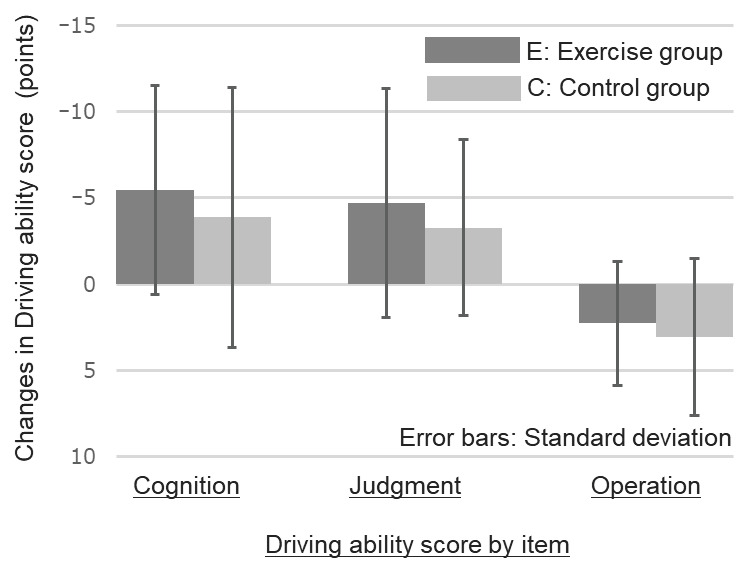
Comparison of changes in driving ability score by item between exercise group and control group.

**Figure 3:**
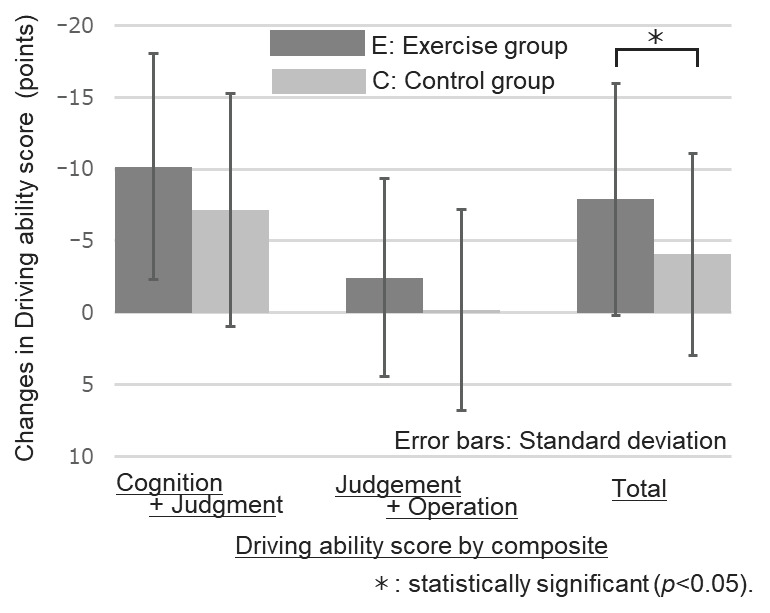
Comparison of changes in driving ability score by composite between exercise group and control group.

The changes before and after the intervention were compared between groups E and C. The results are summarized in Table 3. Figure 2 compares the amount of change in the primary evaluation item and the driving ability evaluation for individual items. A comparison of the overall assessments is shown in Figure 3. No significant differences were observed in individual items regarding the amount of change in driving ability. However, the overall assessment of “Cognition” + “Judgment” + “Operation” revealed a significant difference, and the E-group demonstrated an improvement in overall driving ability compared to the C-group (*p* = 0.02). The mean change in driving ability ratings for the healthy exercise intervention group was −7.89 (SD = 8.07), a clinically meaningful difference.

Differences in the ratio of male to female dropouts caused differences in the proportion of male-to-female dropouts between the groups (not significant in tests of independence: chi-squared test). Therefore, sex and age were used as covariates, and an analysis of covariance was used to compare changes in driving ability ratings. No significant differences were found for the individual items. Still, as in the unadjusted case, we found significant differences only for the overall judgment of driving ability (*p* = 0.03).

## 4. Discussion

### 4.1. Improvement of Driving Ability

This study aimed to investigate the potential of maintaining or improving driving ability through practicing healthy exercises and physical activities among older community members facing driving ability decline. Twelve weeks of healthy exercise practice resulted in an improvement in overall driving ability assessment scores. These findings indicate that appropriate health exercise practices positively affect the maintenance or improvement of driving abilities. However, there were no significant differences in the advancements of the individual evaluation components of “cognition”, “judgment”, and “operation”, respectively.

Furthermore, we compared and examined the results of TMT measurements measured in this study with those of a previous study using TMT measurements in healthy older participants [35,36,37]. No abnormal values were found in the TMT-A or TMT-B measurements of the participants in this study. Thus, the study participants were healthy older adults as regards attention and executive functions.

Kamide et al. examined the relationship between physical function and driving ability among older drivers in 48 adults under the age of 65 (mature and middle-aged groups) and 18 older drivers aged over 65 years, and reported that physical function was independently related to cognitive function in the driving ability of older drivers [38]. Kose et al. surveyed 523 habitual drivers regarding physical function tests and driving anxiety. Their findings indicated that older drivers with driving anxiety had notably lower levels of physical fitness than those without anxiety [39]. These observations reveal a connection between driving ability and physical function, and between driving anxiety and physical function. However, these reports were cross-sectional studies, and the possibility of maintaining or improving driving ability in older adults by improving physical function or increasing physical activity remains to be clarified.

Additionally, healthy exercise among older adults has been reported to have various positive effects on health indicators among older adults. Several direct positive effects of physical fitness indicators have been reported. Filipe et al. reported that regular health exercises for 24 weeks safely and effectively improved functional fitness in community-dwelling older adults [40]. Middleton et al. found that implementing a group physical activity program conserved and enhanced physical health indicators in older adults [41]. Carta et al. reported that healthy exercise and physical activity may improve cognitive function in older adults [42].

This study was based on the following hypothesis: healthy exercise and physical activity among older adults in the community will improve physical function, especially physical fitness, and maintain or improve driving ability. The 12-week intervention of practicing healthy exercise and physical activity significantly improved some physical functions and physical fitness compared to the control group C. It was also confirmed that overall driving ability was significantly improved compared to that of group C. Thus, healthy exercise and physical activity practices may be effective for maintaining and improving older adults’ driving abilities.

Several studies have examined the relationship between motor skills and driving ability. Chen et al. investigated the functional range of motion of the neck and the history of driving crashes [43]. They reported the possibility of assessing driving ability based on the functional range of motion in driving posture. Evrim et al. examined the age-related decline in driving ability from the perspective of physical activity [44]. They reported that cardiorespiratory fitness was associated with the effects of aging on driving ability. Huisingh et al. showed that frequent falls among older drivers are implicated in negligent motor vehicle crashes [45]. This suggests that certain physical functions may be associated with maintaining and improving driving abilities.

In the current intervention, healthy exercise and physical activity practices significantly improved two items within the E-group compared to the C-group: the one-leg open-eye balance test and the 30 s chair standing up test. These specific evaluations address the body’s static and dynamic balance abilities [25,27]. However, it is not clear from the protocol of this study whether the improvement in these items directly affects the maintenance and improvement of driving ability. Thus, factors directly related to the maintenance and improvement of driving ability remain unclear.

Karthaus et al. described driving as requiring the interaction of sensory, motor, and cognitive functions and reported a methodology for assessing driving ability from the perspective of age-related changes in physical functions [46]. A clear identification of factors related to driving ability is essential. The 12-week intervention in this study targeted general improvements in physical function but failed to target detailed physical function items. The potential for more effective health exercises and physical activity interventions could be unlocked with a clearer understanding of the associations between specific physical function items and driving ability. Future intervention studies should focus on targeting more segmented physical function items.

However, there have been many recent reports on social frailty in older adults [47,48,49]. Mehrabi et al. investigated the impact of social isolation on frailty and reported the importance of public health policies regarding social relationships in improving frailty [50]. Motorized transportation holds a pivotal role in fostering social connections, as underscored in this study. Amagasa et al. revealed that older drivers were more physically active than their non-driving counterparts, implying that older drivers can continue to engage in social activities [51]. Driving is an essential means of transportation for individuals, families, and groups, particularly in areas with poor public transportation systems [10]. These facts confirm that securing mobility by driving a car is necessary for building social relationships, supporting and ensuring physical activity, and contributing to improved physical function, all of which may create a positive frailty cycle (frailty prevention).

The need to provide opportunities for human interaction and social participation has been reported even in situations where interaction with others has become difficult owing to the new coronavirus infection [52,53]. The perspective of maintaining continued motorized driving is also necessary for interactions with people in the post-coronavirus period. Maintaining driving ability through healthy exercise is not merely important for transportation. It is also related to the prevention of social frailty and can potentially improve older adults’ health-related quality of life.

Healthy exercise and physical activity are not only effective in directly maintaining and improving physical function and fitness. From the perspective of maintaining and improving driving ability, they can lead to continued social interactions and social participation behaviors in older adults. Driving ability maintained through healthy exercise is not merely a means of transportation, but is also related to the prevention of social frailty and has the potential to contribute to improving health-related quality of life in older adults.

One fundamental future perspective is to establish a more practical and clinical methodology for maintaining and improving driving ability among older adults by clarifying the effects of health exercises and combined interventions.

### 4.2. Methods of Measuring Driving Ability

This study was conducted without using a driving simulator or examiner evaluation. After consideration and deliberation, the examiner’s evaluation was employed; however, some points still needed to be considered. Lee et al. compared driving performance in a driving simulator and on-road driving, showing a significant positive correlation [54]. Groeger et al. conducted a comprehensive assessment of the validity and reliability of driving simulators by comparing multiple driving simulators with actual driving conditions [55]. Furthermore, there has been notable research on the use of driving simulators to evaluate driving performance under various conditions. Azuma et al. used a driving simulator to measure driving performance under conditions of driving under the influence of alcohol and talking while using a Keitai phone [56]. Nonetheless, in a systematic review of 44 studies, Wynne et al. highlighted that approximately one-third of the articles rejected the validity of driving simulators and advocated guidelines for driving simulators in future research [57].

Negative aspects of driving simulators have also been reported. Matas et al. reported that older adults are at a higher risk for driving simulator sickness. Moreover, they found that being female and having a history of motion sickness were associated with driving simulator sickness [58].

In the present study, we evaluated driving ability on the road by an examiner to prevent motion sickness symptoms, as measured by a simulator, because this study was conducted on older participants. The possibility of performing measurements under more realistic conditions was also investigated. In this study, no comparison was made between the evaluations by the simulators and examiners. As automobile driving techniques continue developing, measurement methods must be re-examined.

### 4.3. Limitations of the Study

This study had a relatively short intervention period of 12 weeks. The social impact of the novel coronavirus infection forced us to postpone the start of the intervention study and shorten the intervention period. The effects of the new coronavirus infection continue to make it challenging to conduct interventional studies. Therefore, it is necessary to further examine the setting of intervention periods based on previous studies. Hence, further research on the effects of long-term interventions is warranted.

Recruitment was challenging due to the social impact of the new coronavirus infection. Undeniably, the study participants were biased by their interest in healthy exercise and maintenance of daily physical activity. Additionally, a few participants wished to discontinue their participation in the health exercise classes because of objections from their family members regarding their continued participation in the classes from the perspective of infection prevention. Regarding the health class operation, we made every effort to take all possible sanitary measures to dispel the participants’ concerns. However, the situation was severe. Again, we felt that conducting intervention research during a novel coronavirus pandemic was difficult.

Regarding the study content, the E-group solely participated in unregulated healthy exercise classes without incorporating any other form of physical activity or exercise. Similarly, the C-group adhered to their regular lifestyle without any exercise or physical activity regulations. For both groups, more accurate results could have been obtained by adding a measurement component, like physical activity and exercise practice during the intervention period, using accelerometers and physical activity questionnaires.

## 5. Conclusions

This study found that actively practicing healthy exercise and physical activity holds promise in preserving and enhancing the overall driving ability of older adults within the community. 

## Figures and Tables

**Table 1:** Characteristics of subjects before randomization.

	All Subjects (*n* = 68)		
	Mean ± SD	Minimum	Maximum
Male, *n* (%)	52 (76.5%)		
Age (year)	74.9 ± 3.8	65	82
Height (cm)	155.3 ± 9.0	139.7	185.0
Body weight (kg)	56.3 ± 10.6	36.6	90.0
BMI (kg/m^2^)	23.3 ± 3.3	17.8	37.5
Grip strength (kg)	23.7 ± 5.2	15.4	40.1
Flexibility (cm)	34.0 ± 9.7	13.0	57.0
One leg with eye opened balance (seconds)	51.1 ± 41.6	1.0	120.0
10 meters walking with obstacles (seconds)	8.3 ± 1.4	6.1	12.2
30 s chair standing up test (times)	20.9 ± 4.7	10.0	32.0
Five times chair standing up test (seconds)	6.6 ± 1.8	4.0	13.5
Timed Up and Go Test (seconds)	6.3 ± 1.1	4.5	9.3
TMT-A: Trail Making Test part A (seconds)	60.3 ± 23.5	38.0	176.0
TMT-B: Trail Making Test part B (seconds)	105.2 ± 45.7	37.0	300.0
Driving ability Cognition	9.0 ± 6.2	0	33
Driving ability Judgment	5.8 ± 5.2	0	23
Driving ability Operation	1.2 ± 2.0	0	8
Driving ability Cognition + Judgment	14.8 ± 7.5	3	34
Driving ability Judgment + Operation	7.0 ± 5.5	0	23
Driving ability Cognition + Judgment + Operation	16.0 ± 7.3	3	34

BMI: body mass index (kg/m^2^); SD: standard deviation.

**Table 2:** Baseline characteristics of subjects.

	E: Exercise Group (*n* = 36)	C: Control Group (*n* = 32)
	Mean ± SD	Mean ± SD
Male, *n* (%)	29 (80.1%)	23 (71.9%)
Age (year)	74.8 ± 3.9	75.0 ± 3.6
Height (cm)	155.6 ± 9.7	155.0 ± 8.0
Body weight (kg)	55.9 ± 12.8	56.8 ± 7.2
BMI (kg/m^2^)	22.9 ± 3.7	23.7 ± 2.7
Grip strength (kg)	23.4 ± 4.6	24.0 ± 5.7
Flexibility (cm)	34.8 ± 9.4	33.0 ± 9.8
One leg with eye opened balance (seconds)	46.5 ± 39.0	56.4 ± 43.1
10 meters walking with obstacles (seconds)	8.3 ± 1.4	8.3 ± 1.5
30 s chair standing up test (times)	21.3 ± 4.0	20.5 ± 5.3
Five times chair standing up test (seconds)	6.4 ± 1.3	6.8 ± 2.1
Timed Up and Go Test (seconds)	6.1 ± 1.0	6.5 ± 1.1
TMT-A: Trail Making Test part A (seconds)	58.5 ± 17.3	62.3 ± 28.5
TMT-B: Trail Making Test part B (seconds)	102.2 ± 37.9	108.5 ± 52.3
Driving ability score Cognition	9.0 ± 5.5	9.0 ± 6.8
Driving ability score Judgment	6.2 ± 5.7	5.4 ± 4.4
Driving ability score Operation	1.3 ± 1.9	1.0 ± 2.0
Driving ability score Cognition + Judgment	15.2 ± 7.3	14.3 ± 7.6
Driving ability score Judgment + Operation	7.5 ± 5.7	6.4 ± 5.1
Driving ability score Total	16.5 ± 7.0	15.4 ± 7.4

BMI: body mass index (kg/m^2^); SD: standard deviation; driving ability score total: driving ability score cognition + judgment + operation.

**Table 3:** Comparison of changes in parameters between the two groups.

	E: Exercise Group (*n* = 36)	C: Control Group (*n* = 32)	
	Mean ± SD	Mean ± SD	*p* Value
ΔGrip strength (kg)	−1.2 ± 2.5	0.2 ± 2.8	0.02
ΔFlexibility (cm)	1.0 ± 6.4	4.9 ± 10.9	0.04
ΔOne leg with eye opened balance (seconds)	15.6 ± 34.4	−4.7 ± 34.0	0.01
Δ10 meters walking with obstacles (seconds)	−0.4 ± 0.9	−0.2 ± 1.0	0.24
Δ30 s chair standing up test (times)	5.7 ± 5.3	−1.1 ± 5.3	<0.001
ΔFive times chair standing up test (seconds)	−0.1 ± 1.3	−0.3 ± 1.3	0.23
ΔTimed Up and Go Test (seconds)	−0.6 ± 0.9	−0.8 ± 0.7	0.12
ΔTMT-A: Trail Making Test part A (seconds)	−16.4 ± 12.3	−19.6 ± 24.4	0.24
ΔTMT-B: Trail Making Test part B (seconds)	−2.7 ± 33.9	2.1 ± 65.0	0.35
ΔDriving ability Cognition	−5.44 ± 6.07	−3.88 ± 7.52	0.17
ΔDriving ability Judgment	−4.72 ± 6.63	−3.28 ± 5.08	0.16
ΔDriving ability Operation	2.28 ± 3.58	3.09 ± 4.55	0.21
ΔDriving ability Cognition + Judgment	−10.17 ± 7.84	−7.16 ± 8.11	0.06
ΔDriving ability Judgment + Operation	−2.44 ± 6.91	−0.19 ± 7.00	0.09
ΔDriving ability Total	−7.89 ± 8.07	−4.06 ± 7.01	0.02

ΔDriving ability total: ΔDriving ability cognition + judgment + operation; SD: standard deviation; Bold values are statistically significant (*p* < 0.05).

## Data Availability

Not applicable.
